# Evaluation of the POSSUM, P-POSSUM and E-PASS scores in the surgical treatment of hilar cholangiocarcinoma

**DOI:** 10.1186/1477-7819-12-191

**Published:** 2014-06-24

**Authors:** Hui Wang, Haolu Wang, Tao Chen, Xiaowen Liang, Yanyan Song, Jian Wang

**Affiliations:** 1 Department of Biliary-Pancreatic Surgery, Ren Ji Hospital, School of Medicine, Shanghai Jiao Tong University, 1630 S. Dongfang Road, Shanghai 200127, China; 2 Therapeutics Research Centre, Princess Alexandra Hospital, School of Medicine, The University of Queensland, Woolloongabba QLD 4102, Australia; 3 Department of Biostatistics, Institutes of Medical Sciences, School of Medicine, Shanghai Jiao Tong University, 280 S. Chongqing Road, Shanghai 200025, China

**Keywords:** POSSUM, P-POSSUM, E-PASS, Morbidity, Mortality, Hilar cholangiocarcinoma

## Abstract

**Background:**

The Physiological and Operative Severity Score for the enUmeration of Mortality and morbidity (POSSUM) model, its Portsmouth (P-POSSUM) modification and the Estimation of physiologic ability and surgical stress (E-PASS) are three surgical risk scoring systems used extensively to predict postoperative morbidity and mortality in general surgery. The aim was to undertake the first study of the predictive value of these models in patients undergoing surgical treatment of hilar cholangiocarcinoma.

**Methods:**

A retrospective analysis was performed on data collected prospectively over a 10-year interval from January 2003 to December 2012. The morbidity and mortality risks were calculated using the POSSUM, P-POSSUM and E-PASS equations.

**Results:**

One hundred patients underwent surgical treatment of hilar cholangiocarcinoma. Complications were seen in 52 of 100 patients (52.0%). There were 10 postoperative in-hospital deaths (10.0%). Of 31 preoperative and intraoperative variables studied, operative type (*P* = 0.000), preoperative serum albumin (*P* = 0.003) and aspartate aminotransferase (*P* = 0.029) were found to be factors multivariate associated with postoperative complications. Intraoperative blood loss (*P* = 0.015), Bismuth-Corlette classification (*P* = 0.033) and preoperative hemoglobin (*P* = 0.041) were independent factors multivariate associated with in-hospital death. The POSSUM system predicted morbidity risk effectively with no significant lack of fit (*P* = 0.488) and an area under the ROC curve (AUC) of 0.843. POSSUM, P-POSSUM and E-PASS scores showed no significant lack of fit in calculating the mortality risk (*P* >0.05) and all yielded an AUC value exceeding 0.8. POSSUM had significantly more accuracy in predicting morbidity after major and major plus operations (O:E (observed/expected) ratio 0.98 and AUC 0.901) than after minor and moderate operations (O:E ratio 1.13 and AUC 0.759).

**Conclusions:**

POSSUM, P-POSSUM and E-PASS scores effectively predict morbidity and mortality in surgical treatment of hilar cholangiocarcinoma. However, improvements are still needed in the future because none of these scoring systems yielded an AUC value exceeding 0.9 for operations with all different levels of severity. Only POSSUM had more accuracy in predicting postoperative morbidity after operations with higher severity.

**Trial registration:**

This study was undertaken after obtaining approval from the ethics committee of School of Medicine, Shanghai Jiao Tong University with a trial registration number of http://09411960800.

## Background

In the last 20 years surgical treatment of hilar cholangiocarcinoma has mainly evolved because of the enhanced appreciation of tumor characteristics and improvements in preoperative imaging [1]. However, due to complex biliary and hepatic resections, this surgical procedure is still considered to be one of the most challenging procedures faced by hepatobiliary surgeons. Postoperative morbidity and mortality rates remain in a high range (14 to 76% and 0 to 19%, respectively) even in reports from high-volume centers [1-5]. The accurate prediction of outcomes after a high-risk procedure such as surgical treatment of hilar cholangiocarcinoma can early detect postoperative complications, allow improved treatment planning and increase the precision of individual prognosis.

Many surgical risk scoring systems have been devised, but the Physiologic and Operative Severity Score for the enUmeration of Mortality and Morbidity (POSSUM) model by Copeland *et al*. [6] was recommended as the most appropriate for general surgery [7,8]. This model, utilizing scores relating to twelve physiological and six operative variables, was developed to predict in-hospital mortality and morbidity postoperatively. However, POSSUM was then reported as over predicting postoperative mortality, particularly in patients at low risk. This led to a revision: the Portsmouth modification (P-POSSUM) by Whiteley *et al*. [9] Another surgical risk scoring system that has been validated in hepatobiliary surgery worldwide is the Estimation of Physiologic Ability and Surgical Stress (E-PASS) [10-12]. This system comprises a preoperative risk score (PRS), a surgical stress score (SSS) and a comprehensive risk score (CRS) that is calculated from both the PRS and SSS.

Surgical risk scoring systems were initially designed for large populations and populations with different pathologies. However, later they were also applied to patients with a single diagnosis or one type of operation. It is unclear whether these two surgical risk scoring systems are useful for predicting morbidity and mortality in high-risk surgery such as surgical treatment of hilar cholangiocarcinoma. Hellmann *et al.*[13] was the first and only study to evaluate POSSUM for the surgical treatment of cholangiocarcinoma, and they found that the model overestimated postoperative morbidity and mortality. However, owing to its heterogeneity in selection design, this study considered only a limited and unclear number of operations for hilar cholangiocarcinoma over a 13-year period. The aim of the present study was to evaluate the POSSUM and E-PASS score in surgery for hilar cholangiocarcinoma, a most high-risk context in which to our knowledge, they have never been applied.

## Methods

### Patients

Data between January 2003 and December 2012 was analyzed retrospectively from a prospectively maintained database. Consecutive patients treated surgically in this center following a diagnosis of hilar cholangiocarcinoma were studied and only patients with histologically confirmed cholangiocarcinoma were included. Patients who underwent liver transplantation were not included in this study because a significant difference was shown in factors associated with morbidity or mortality compared with other types of operation.

To evaluate the different treatment strategies during the years, these 10 years were divided into three periods in time. Operative technique and postoperative care have been developed during the 10 years and the last period (2009 to 2012) was characterized by more extensive resections. The operative approach in the last four years was performed as described below [14,15]. Generally, in patients with Bismuth-Corlette classification type I, we performed bile duct resection only. In patients with types II and III, an (extended) right or left hepatectomy was performed. Patients with type IV underwent a right or left trisectionectomy. We routinely dissected the lymph nodes surrounding the hepatoduodenal ligament, behind the pancreatic head and around the common hepatic artery. Biliary continuity was achieved by Roux-en-y hepaticojejunostomy with an isoperistaltic 70 cm limb of jejunum. Patients underwent exploratory laparotomy with palliative biliodigestive anastomosis or without curative intent after the surgeon found unresectable hilar cholangiocarcinoma.

All preoperative, intraoperative and postoperative patient data (31 preoperative and intraoperative variables) were collected and entered into a computer database prospectively. The POSSUM, E-PASS scoring systems and multivariate analysis were done retrospectively from the collected data and medical records according to defined criteria. The morbidity risk was calculated using the POSSUM equation. The mortality risk was calculated using the POSSUM, P-POSSUM and E-PASS equations respectively. Complication was evaluated based on the original POSSUM [6] and E-PASS [16] definitions and graded according to the Clavien complication scheme [17]. The in-hospital mortality was recorded for each patient.

### Statistical analysis

Clinical parameters were tested using the *χ*2 goodness-of-fit for comparison within the three time periods. Multivariate analysis was performed using the logistic regression method. Frequency tables were constructed with 10 risk bands and compared with the *χ*2 test using the methods of Hosmer and Lemeshow to test the goodness of fit [18]. A good model is indicated by a high *P* value. In order to predicted postoperative morbidity or mortality rate from the lowest to the highest risk in each model the 10 risk bands were divided. Each risk band contained the same number of subjects. Expected and observed complications or deaths were quantified in each band.

The discriminatory power of each model was assessed by calculating the area under receiver-operating characteristic curve (AUC). Values ranging from 0.7 to 0.9 represent reasonable discrimination. Values exceeding 0.9 represent good discrimination. The differences in morbidity and mortality rate between the risk bands were analyzed using the *χ*2 goodness-of-fit test. Categorical variables were compared between groups using the *χ*2 test with Yates’s correction for continuity [11]. Statistical calculations were carried out with SPSS computer software (SPSS, Chicago, Illinois, United States). A value of *P* <0.05 was considered statistically significant.

## Results

### Parameters and outcome

The 100 consecutive patients who underwent surgical treatment of hilar cholangiocarcinoma during the study period were included in the present study (Table 1). Four of the patients had hepatolithiasis and one had liver cirrhosis. Thirteen of the patients had diabetes, five had hypertension and six had coronary disease. The preoperative serum concentration of total bilirubin was greater than 18 umol/L in 96 of the patients. The preoperative hemoglobin was less than 10 g/dl in 13 of the patients. Ten of the patients had Child’s grade A, 85 had Child’s grade B and 5 had Child’s grade C liver status. The various types of operation performed are shown in Table 2. Since the second period (2006 to 2008), our center adopted new aggressive approaches for patients with hilar cholangiocarcinoma, which resulted in a R0 resection rate increase from 21% in the first period to 45% in the last period (Table 3). Postoperative complications were seen in 52 of 100 patients (52.0%), with some patients having more than one complication. There were 10 in-hospital deaths (10%). The postoperative morbidity and in-hospital mortality were not different among the three periods (*P* >0.05).

**Table 1:** Characteristics of patients undergoing surgery for hilar cholangiocarcinoma

	**100 patients**
Age (mean)	62.9 years
Male gender (%)	48 (48.0%)
AJCC stage I/II/III/IV	7/15/17/61
Bismuth classification I/II/IIIa/IIIb/IV	27/13/21/12/27
R0 Resection	34
R1 Resection	12
R2 Resection	16
Palliative biliodigestive anastomosis	31
Exploration (without curative intent)	7
Overall complications (%)	52 (52.0%)
Pneumonia	19
Bile leak	18
Pleural effusion	9
Hemoperitoneum	3
Wound infection	5
Gastrointestinal bleeding	4
Intra-abdominal abscess	4
Bile duct infection	4
Miscellaneous	4
Liver failure	3
Urinary tract infections	1
Biliodigestive leakage	1
Others (DIC, cerebral infarction)	2
In-hospital mortality (%)	10 (10.0%)

AJCC: American Joint Committee on Cancer; DIC: disseminated intravascular coagulation; R0 resection: complete resection with microscopic examination of margins showing no tumor cells; R1 resection: complete resection with no grossly visible tumor, but examination of margins showing tumor cells; R2 resection: partial resection, with grossly visible tumor left.

**Table 2:** Type of operation performed in 100 patients with hilar cholangiocarcinoma

**Bismuth classification**						**Operative severity**
	**I**	**II**	**IIIA**	**IIIB**	**IV**	
Exploration (without curative intend)	1	1	0	1	4	Minor
Bypass operation (unresectable)	13	6	5	3	4	Moderate
Bile duct resection	13	6	6	2	12	Major
Perihilar hepatectomy	0	0	1	0	0	Major
Right hemihepatectomy	0	0	7	0	3	Major
Left hemihepatectomy	0	0	0	5	3	Major
Extended right hemihepatectomy*	0	0	2	0	0	Major+
Extended left hemihepatectomy*	0	0	0	1	1	Major+

*Combined with resection of segment 1.

**Table 3:** R0 resection, postoperative morbidity and in-hospital mortality in relation to the different periods

**Period**	**2003-2005**	**2006-2008**	**2009-2012**	**Total**	***P***
Number of patients	19	37	44	100	
R0 resection	4 (21%)	11 (27%)	20 (45%)	34 (34%)	0.091
Morbidity	13 (68%)	15 (41%)	24 (55%)	52 (52%)	0.128
Mortality	4 (21%)	3 (8%)	3 (7%)	10 (10%)	0.255

R0 resection: complete resection with microscopic examination of margins showing no tumor cells.

### Multivariate analysis of factors associated with morbidity or mortality

The thirty-one factors included in univariate analysis for postoperative morbidity or mortality were: sex, age, duration of disease, history of operation, pulse, blood pressure, Child’s grade, ASA (American Society of Anaesthesiologists) classification, Glasgow Coma score, cardiopulmonary diseases, diabetes mellitus, hepatolithiasis, preoperative temperature, preoperative serum sodium, potassium, preoperative total bilirubin, white blood cell count, hemoglobin, prothrombin time, aspartate aminotransferase, alanine aminotransferase, γ-glutamyl transpeptidase, albumin, preoperative biliary drainage, operative type, operative duration, intraoperative blood loss, blood transfusion, vascular resection, TNM (tumor, lymph nodes and metastasis) stage and Bismuth-Corlette classification.

The significant factors determined by univariate analysis for postoperative morbidity were: vascular resection (*P* = 0.018), preoperative serum albumin (*P* = 0.019), operative type (*P* = 0.000), operative duration (*P* = 0.000), intraoperative blood loss (*P* = 0.003), blood transfusion (*P* = 0.001) and Bismuth-Corlette classification (*P* = 0.017). Operative type (*P* = 0.000), preoperative serum albumin (*P* = 0.003) and aspartate aminotransferase (*P* = 0.029) were found to be factors multivariate associated with postoperative complications (Table 3).

The significant factors determined by univariate analysis for in-hospital mortality were: preoperative serum hemoglobin (*P* = 0.007), vascular resection (*P* = 0.049), operation type (*P* = 0.019), intraoperative blood loss (*P* = 0.001), blood transfusion (*P* = 0.006) and Bismuth-Corlette classification (*P* = 0.014). Intraoperative blood loss (*P* = 0.015), Bismuth-Corlette classification (*P* = 0.033) and preoperative hemoglobin (*P* = 0.041) were found to be the only independent factor multivariate associated with in-hospital death (Table 4).

**Table 4:** Significant multivariate associations between study variables and postoperative morbidity and in-hospital mortality

	**OR**	**95% CI**	***P***
**Morbidity**			
Operation type	6.661	3.058-14.509	0.000
Preoperative serum albumin, mg/dL	0.194	0.065-0.583	0.003
Aspartate aminotransferase (AST), U/L	10.304	1.268-83.754	0.029
**Mortality**			
Intraoperative blood loss, ml	3.259	1.262-8.416	0.015
Bismuth classification	0.154	0.028-0.859	0.033
Preoperative hemoglobin, g/l	1.973	1.028-3.787	0.041

CI: confidence interval; OR: odds ratio.

### POSSUM, P-POSSUM and E-PASS scores

**Figure 1:**
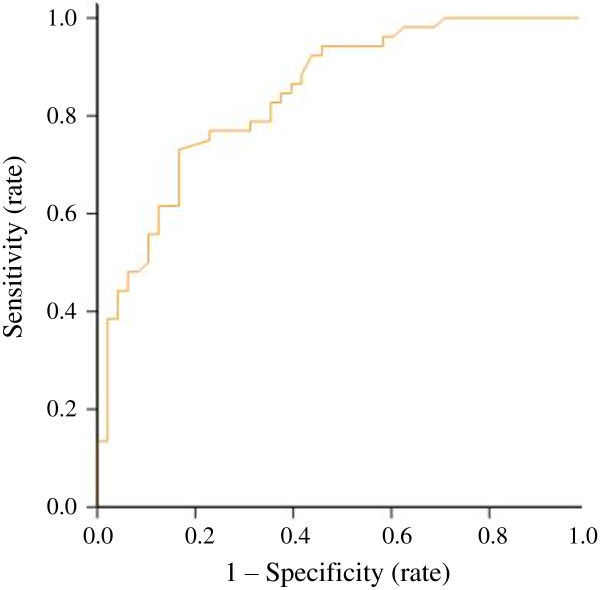
**ROC curve for POSSUM predicting postoperative morbidity in surgical treatment of hilar cholangiocarcinoma.** ROC curve: receiver operating characteristic curve; POSSUM: Physiological and Operative Severity Score for the enUmeration of Mortality and morbidity.

**Figure 2:**
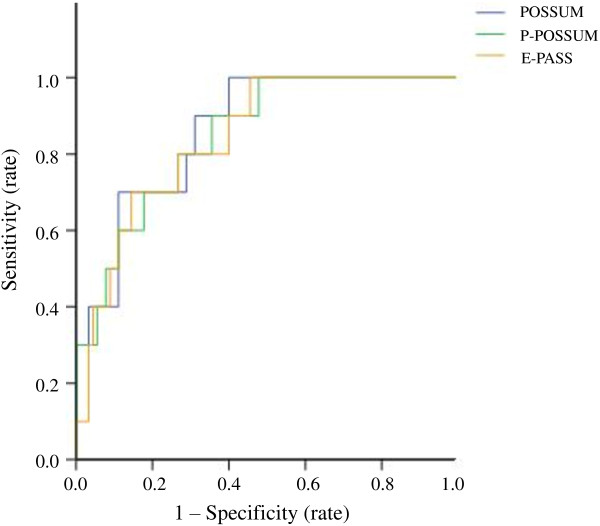
**ROC curve for POSSUM, P-POSSUM and E-PASS scores predicting in-hospital mortality in surgical treatment of hilar cholangiocarcinoma.** ROC curve: receiver operating characteristic curve; POSSUM: Physiological and Operative Severity Score for the enUmeration of Mortality and morbidity; P-POSSUM: Portsmouth modification of POSSUM; E-PASS: Estimation of physiologic ability and surgical stress.

The calibration power of POSSUM, P-POSSUM and E-PASS was analyzed using the Hosmer-Lemeshow test after 10 risk bands were divided (Table 5 and Table 6) [18]. Statistically significant differences were detected in the postoperative morbidity or in-hospital mortality rate between the risk bands of all three models using the *χ*2 goodness-of-fit test. When comparing predicted morbidity with observed morbidity by POSSUM score, an overall O:E (observed/expected) ratio of 1.00 was found (Table 7). This model showed no significant lack of fit (*P* = 0.488) and yielded an AUC of 0.843 (Figure 1). The POSSUM, P-POSSUM and E-PASS scores showed no significant lack of fit in calculating the mortality risk (*P* >0.05). P-POSSUM and E-PASS performed well and gave an O:E ratio of 1.00, while POSSUM gave an O:E ratio of 1.11 (Table 7). All scoring systems yielded an AUC value exceeding 0.8 and none of them showed a higher AUC value in predicting in-hospital mortality than the others (*P* 0.05, Table 7 and Figure 2).

**Table 5:** Calibration power of POSSUM score for predicting postoperative morbidity

	**POSSUM**	
**Risk band**	**E**	**O**
1	1	0
2	1	2
3	2	2
4	4	6
5	4	3
6	6	7
7	8	7
8	8	7
9	10	10
10	8	8
Total	52	52

POSSUM: Physiological and Operative Severity Score for the enUmeration of Mortality and morbidity; E: expected; O: observed.

**Table 6:** Calibration power of POSSUM score for predicting postoperative morbidity

	**POSSUM**		**P-POSSUM**		**E-PASS**	
**Risk band**	**E**	**O**	**E**	**O**	**E**	**O**
1	0	0	0	0	1	0
2	0	0	1	0	0	0
3	0	0	0	0	0	0
4	0	0	0	0	1	1
5	0	0	1	1	1	1
6	0	1	1	1	1	1
7	1	2	1	1	1	2
8	1	0	1	1	2	3
9	2	3	2	3	3	2
10	5	4	4	3	-	-
Total	9	10	10	10	10	10

POSSUM: Physiological and Operative Severity Score for the enUmeration of Mortality and morbidity; P-POSSUM: Portsmouth modification of POSSUM; E-PASS: Estimation of physiologic ability and surgical stress; E: expected; O: observed.

**Table 7:** Predictive value of four surgical risk scoring systems of postoperative morbidity and in-hospital mortality

	**O/E ratio**	***P*** *	AUC** (95% CI)
Morbidity			
POSSUM	1.00 (52/52)	0.488	0.843 (0.768-0.919)
Mortality			
POSSUM	1.11 (10/9)	0.520	0.863 (0.766-0.961)
P-POSSUM	1.00 (10/10)	0.721	0.848 (0.740-0.956)
E-PASS	1.00 (10/10)	0.671	0.842 (0.735-0.949)

*: goodness-of-fit analysis.

**: receiver operating characteristic curve.

POSSUM: Physiological and Operative Severity Score for the enUmeration of Mortality and morbidity; P-POSSUM: Portsmouth modification of POSSUM; E-PASS: Estimation of physiologic ability and surgical stress.

To evaluate the effect of different surgical procedures on the predictive value of each model, all cases were divided into four groups according to the operative severity. Definitions of the operative severity were shown in Table 2. POSSUM had significantly more accuracy in predicting morbidity after major and major plus operations (O:E ratio 0.98 and AUC 0.901) than after minor and moderate operations (O:E ratio 1.13 and AUC 0.759, *P* <0.05). However, no additional value was found for POSSUM, P-POSSUM and E-PASS scores in predicting in-hospital mortality after major and major plus operations (O:E ratio 1.33, 1.14, 1.33 and AUC 0.803, 0.796, 0.852) compared to those after minor and moderate operations (O:E ratio 1.00, 1.00, 0.00 and AUC 0.986, 0.973, 0.833, *P* >0.05).

## Discussion

Postoperative complications and death may result depending on three major factors: the quality of the surgical team, the patient’s physiological status and the degree of surgical stress [16]. Where the quality of a surgical team in one hospital has remained stable for a certain period, surgical risk scoring systems could be applied to assess the risk of complications and death by quantification of the patient’s physiological status and the surgical stress applied. Several surgical groups have used POSSUM [17], P-POSSUM [18] and E-PASS [12,19] successfully to perform comparative audit in hepato-biliary-pancreatic surgery. Therefore, these two scoring systems were chosen in the present study.

This is the first time surgical risk scoring systems were applied to a specific hilar cholangiocarcinoma surgical patient population. With a postoperative morbidity rate of 52.0% and an in-hospital mortality rate of 10.0%, our institution lies within the accepted range of complications after surgical treatment of hilar cholangiocarcinoma [1]. When the *χ*2 test was used to compare actual morbidity and mortality rates with estimated ones, there was no significant lack of fit (*P* >0.05), indicating that the POSSUM and E-PASS scoring systems accurately estimate the outcomes. They also yielded an AUC value exceeding 0.7, suggesting their utility in predicting morbidity and mortality after surgery for hilar cholangiocarcinoma. However, improvements are still needed in the future because none of these scoring systems yielded an AUC value exceeding 0.9 for operations with all different levels of severity. Previous meta-analysis [20] and some reports [10] revealed that the POSSUM and E-PASS scoring systems failed to offer a significant predictive value for morbidity and mortality after hepatobiliary surgery. The main reason for the different findings may be that surgery of hilar cholangiocarcinoma is a more complex and severe operation than other hepatobiliary procedures. It has a higher operative severity score in POSSUM and a higher surgical stress score in E-PASS and therefore results in a higher risk prediction. Because the potential for morbidity and mortality is greater after this operation, surgical risk scoring systems would demonstrate a more accurate predictive value. We evaluated the corresponding results if only the patients underwent major and major plus operations were included. POSSUM indeed had more accuracy in predicting postoperative morbidity after major and major plus + operations. Similar findings have been observed in other studies, where POSSUM has a significantly more accurate predictive value for higher acuity procedures, such as pancreaticoduodenectomy, than for other pancreatic surgeries [17,21]. However, no additional value was found for POSSUM, P-POSSUM and E-PASS scores in predicting in-hospital mortality after major and major plus operations. Firstly, operative type was not a factor multivariate associated with postoperative complications in our study, therefore, it remains unclear whether the type of operation influences the validity of the scores. Secondly, some independent factors for morbidity and mortality, such as operation type, intraoperative blood loss and preoperative hemoglobin are scored in POSSUM, P-POSSUM and E-PASS. Multivariate predictors for hepatobiliary surgery may differ from those in POSSUM and E-PASS scoring systems [22]. Based on the findings of our multivariate analysis, preoperative serum albumin, aspartate aminotransferase, and the Bismuth classification are independent factors associated with postoperative morbidity or in-hospital mortality but are included neither in E-PASS nor in POSSUM systems. Therefore, if researchers would like to improve the AUC value of these surgical scoring systems for hilar cholangiocarcinoma in the future, these factors might be added as new parameters in revised models.

Among the three surgical risk scoring systems employed in the present study, none of them showed a higher AUC value in predicting in-hospital mortality than the others. The advantage of the E-PASS scoring system is the relative ease with which data are acquired. This is favorable to the POSSUM or P-POSSUM score, which requires 18 different variables compared with the nine variables needed for the E-PASS score [23]. Furthermore, the POSSUM was generated only for surgical auditing and not for surgical decision making. However, the application of E-PASS has a potential role not only in surgical auditing but also in surgical decision making both between and within individual practice [24]. In our institution, we have developed preoperative management scenarios in our pancreato-biliary surgical practice. For example, patients with a high comprehensive risk score (CRS) are provided additional preoperative interventions such as enteric tube feedings, hyperalimentation, antibiotics, and biliary stenting, to improve preoperative parameters. This is often indicated, particularly when patients present with malignant obstructive jaundice, comorbid cardiac or respiratory illness, diabetes, or malnutrition.

There are some limitations to the present study. Firstly, since hilar cholangiocarcinoma is an uncommon neoplasm, the mortality rate corresponds to only ten patients, resulting in a relatively small group (100 patients) available for analysis over a long period of time (2003 to 2012). Operative technique and postoperative care have been developed during the past 10 years and treatment strategies are evolving. Secondly, in constructing the E-PASS model postoperative complications were only included when medical or interventional treatment had been carried out and mild complications were not regarded to be the same as severe ones [16]. However, POSSUM and P-POSSUM use a different definition and analysis of complication [6] which may affect the comparison of predictive value of scoring systems in our study.

## Conclusions

The present study shows that POSSUM, P-POSSUM and E-PASS scores effectively predict morbidity and mortality in surgical treatment of hilar cholangiocarcinoma. However, improvements are still needed in the future because none of these scoring systems yielded an AUC value exceeding 0.99 for operations with all different levels of severity. Only POSSUM had more accuracy in predicting postoperative morbidity after operations with higher severity.

## Abbreviations

AJCC: American Joint Committee on Cancer; AUC: Area under receiver operating characteristic curve; CRS: Comprehensive risk score; DIC: disseminated intravascular coagulation; E-PASS: Estimation of physiologic ability and surgical stress; POSSUM: Physiological and Operative Severity Score for the enUmeration of Mortality and morbidity; P-POSSUM: Portsmouth modification of POSSOM; PRS: Preoperative risk score; SSS: A surgical stress score.; ROC curve: receiver operating characteristic curve.

## Competing interests

The authors declare that they have no competing interests.

## Authors’ contributions

HuW, HaW and TC carried out data acquisition. HaW and JW conceived the project and designed the study. HuW and HaW drafted the manuscript. TC, YS and XL carried out statistical analyses. HaW and XL revised the manuscript. All authors read and approved the final manuscript.
